# P2RX1-Involved Glycolytic Metabolism Supports Neutrophil Activation in Acute Pancreatitis

**DOI:** 10.3389/fimmu.2020.549179

**Published:** 2021-02-02

**Authors:** Xu Wang, Dadong Liu, Weiting Qin, Yishu Liu, Xiao Yuan, Xiaoxin Zhang, Chunhua Dai, Danyi Zhang

**Affiliations:** ^1^ Department of Radiation Oncology, Institute of Oncology, Affiliated Hospital of Jiangsu University, Zhenjiang, China; ^2^ Department of Intensive Care Units, Affiliated Hospital of Jiangsu University, Zhenjiang, China; ^3^ Department of Radiation Oncology, Renji Hospital, School of Medicine, Shanghai Jiao Tong University, Shanghai, China; ^4^ Department of General Surgery, Affiliated Hospital of Jiangsu University, Zhenjiang, China; ^5^ Jiangsu Key Laboratory of Medical Science and Laboratory Medicine, School of Medicine, Jiangsu University, Zhenjiang, China; ^6^ Department of Laboratory Medicine, Affiliated People’s Hospital of Jiangsu University, Zhenjiang, China

**Keywords:** acute pancreatitis, inflammation, neutrophil, purinergic signaling, purinergic receptor

## Abstract

Acute pancreatitis (AP) is characterized by disordered inflammation of the pancreas, and the underlying mechanisms remain unclear. Purinergic signaling plays crucial roles in initiating and amplifying inflammatory signals. Recent evidence reveals that targeting dysregulated purinergic signaling is promising for treating inflammation-associated diseases. To explore the potential involvement of purinergic signaling in AP, we investigated the expression profiles of purinergic signaling molecules in human and mouse pancreas tissues. Results showed that purinergic receptor P2RX1 was among the most highly expressed genes in both human and mouse pancreas tissues. Genetic ablation or specific antagonism of P2RX1 markedly alleviated inflammatory responses in caerulein-induced AP mice. Bone marrow chimeras and adoptive transfer studies revealed that neutrophil-derived P2RX1 contributed to the inflammatory responses in AP. Further studies demonstrated that P2RX1 promoted neutrophil activation by facilitating glycolytic metabolism. Therefore, our study indicates that purinergic receptor P2RX1 may be a potential therapeutic target to treat disordered inflammation in AP.

## Introduction

Acute pancreatitis (AP) is a necroinflammatory disease of pancreas tissues, which is associated with a mortality rate of 20% in its severe form (1). Due to the lack of specific therapy, the morbidity and mortality of AP still remain at a high level (2). The etiological factors involved in the initiation and aggravation of disordered inflammation in AP are poorly understood (3, 4). Therefore, efforts to understand the pathogenesis and develop novel strategies to attenuate the disease severity are imperative.

Neutrophils are the most abundant immune cells in the human blood. In the early phase of AP, neutrophils are considered as the first responder cells which are recruited to pancreas tissues and contribute to the initiation of disordered inflammation (5). Targeting neutrophils by cell depletion strategy was shown to improve the prognosis of AP in a murine model (6). However, ablating all neutrophils is not a clinically feasible strategy due to their essential roles in antimicrobial immunity (7). Understanding how neutrophils are substantially activated in AP and finding specific targets are crucial for improving the present neutrophil depletion strategy.

Nucleotides (particularly ATP) and nucleosides (ADO) are the basic elements of all living organisms. Purinergic signaling, which utilizes extracellular nucleotides and nucleosides as signaling transduction molecules, is an evolutionarily selected system that fine-tunes various cell functions (8). Emerging evidence uncovers that targeting purinergic signaling represents a novel approach to modulating immune disorders (9). However, until now, little is known about the specific roles of purinergic signaling in AP. Therefore, the present study investigated the expression profiles of purinergic signaling receptors in pancreas tissues and found that purinergic receptor P2RX1 was among the most highly expressed genes. Bone marrow chimeras and neutrophil adoptive transfer studies revealed that neutrophil-derived P2RX1 accounted for the progression of AP. Further studies showed that P2RX1-induced enhanced glycolytic metabolism contributed to neutrophil activation.

## Materials and Methods

### Animal Models

With the help from GemPharmatech Co., Ltd., global P2RX1 knockout (KO) mice were constructed in the C57BL/6 background with CRISPR/Cas9 system. Guide RNA sequences targeting Exon2–Exon3 of P2rx1 gene were: S1: CATCCAACGACGCAAGTGGC (PAM: TGG); S2: TGGTTCGCAAGTAGTTTCCC (PAM: AGG); S3: ATGGTGCTGTTGCGGGGCAC (PAM: TGG); S4: TTGGTCCTTGGTGGTAATGT (PAM: GGG). Mice were housed in the same room in a specific pathogen-free facility under a 12-h light/dark cycle with free access to food and water. To induce acute pancreatitis, male mice (6–8 weeks old) were intraperitoneally injected with caerulein (50 μg/kg; Sigma-Aldrich) seven times hourly. Apyrase (50 U/kg, Sigma), PPADS (5 mg/kg, Tocris), and NF449 (5 mg/kg, Tocris) were intraperitoneally injected immediately after the first injection of caerulein. Mice were euthanatized at indicated time points to obtain blood and pancreas tissues. Saline-treated mice were euthanatized at 8-h and set as negative control (NC).

### Bone Marrow Transplantation

Bone marrow transplantation was performed as previously reported (10). Briefly, 6–8 weeks old mice were used for bone marrow translation studies. Recipient mice were exposed to lethal irradiation (11 Gy) and subsequently received intravenous injection of 5 × 10^6^ donor bone marrow cells. After 6–8 weeks, bone marrow chimeric mice were obtained and used for induction of AP.

### Neutrophil Isolation and Stimulation

Bone marrow neutrophils isolation were performed as previously reported (11). Briefly, bone marrow cells were flushed from mouse femur/tibia bones and resuspended in HBSS without calcium and magnesium. Centrifuging through a discontinuous Percoll gradient (78, 69, and 52%), bone marrow neutrophils were harvested between the 78 and 69% layers. For neutrophil stimulation, serum from WT caerulein-treated AP mice or vehicle-treated control mice was obtained at 8 h after first dosage of caerulein and filtered through 0.22 μm micronylon filter. Neutrophils were stimulated with 30% AP serum in RMPI 1640 culture medium. Flow cytometry cell sorting was performed to isolate neutrophils from pancreas tissues. Briefly, tissues were carefully minced and digested with 2 mg/ml collagenase A (Sigma) and 1× DNase I (Sigma) for 30 min. Digestion was quenched by fetal bovine serum (FBS) and filtered with 70 μm Nylon mesh. PE-labeled anti-Ly6G antibody (1:100, Biolegend) and APC-labeled anti-CD45 antibody (1:100 Biolegend) were co-stained to gate and sort neutrophils.

### Neutrophil Depletion and Adoptive Transfer

Neutrophil depletion and adoptive transfer experiments were performed as previously reported (12). Briefly, neutrophils in WT mice were depleted by an intraperitoneal injection of 300 μg anti-Ly6G antibody (1A8, Biolegend) 24 h before the first administration of caerulein. 2.5 × 10^6^/ml neutrophils isolated from bone marrow of WT or P2RX1-KO mice were resuspended in 200 μl PBS and intravenously injected 1 h before the first administration of caerulein.

### Biochemical Assays

ATP concentration was detected using an ATP assay kit following the manufacturer’s instructions (Abcam). Briefly, blood was collected by heart punctures and centrifuged to obtain plasma. ATP was detected fluorometrically (Ex/Em = 535/587 nm) and quantified according to standard curve. Serum amylase activity was detected photometrically with an automated analyzer from Roche. Pancreas tissues were harvested from each group and tissue homogenates were prepared on ice. MDA (Sigma) and MPO (Sigma) assay kits were used to evaluate inflammatory status according to the manufacturer’s instructions.

### Pancreas Edema and Morphology Analyses

Pancreas tissues were harvested at indicated time points and weighted to measure wet weight. After desiccation at 160°C for 12 s, pancreas tissues were weighted to measure dry weight. Then wet/dry weight ratio was calculated. For morphology study, pancreas tissues were harvested at 8 h after fist dosage of caerulein and fixed with 2% paraformaldehyde immediately. Sections (2 μm) were stained with H&E routinely.

### Metabolic Assay

Seahorse XF96 Flux Analyser (Agilent) was applied to measure extracellular acid ratio (ECAR) according to the manufacturer’s instructions. Briefly, neutrophils were stimulated with AP serum for 6 h and then harvested. For ECAR test, 1 μmol/L oligomycin, 0.5 μmol/L carbonyl cyanide p-trifluoromethoxyphenylhydrazone (FCCP), and 0.5 μmol/L rotenone plus 0.1 μmol/L antimycin A were injected to the wells.

### RT-qPCR

Pancreas tissues were harvested at indicated time points and neutrophils were harvested 6 h after stimulation with AT serum. Samples were immediately frozen in liquid nitrogen after collection to avoid RNA degradation. 1 ml TRIzol (Thermo) per 20 mg wet weight of pancreas was used to isolate total RNAs. RNA purity and integrity were then determined by Thermo Nanodrop 2000 and Agilent 2100 Bioanalyzer, respectively. A260/A280 greater than 1.8 and RNA integrity number (RIN) ratio greater than seven were considered as qualified samples. Real-time PCR analyses were applied for gene expression study, and SYBR Premix Ex Taq (Roche) was used to run PCR on a 7500 Real-time PCR system (Applied Biosystems) at the recommended thermal settings. The relative gene expression value of purinergic signaling molecules was calculated by the comparative C_t_ method: 2^−(indicated gene Ct value −^ *^β^* ^-action Ct value)^ × 100 as previously reported (13). Quantitative 2^−^ *^δδ^* ^Ct^ value was used for comparing the fold change of inflammatory cytokines.

The following primers were used: IL-1*β*-F: CACGATGCACCTGTACGATCA, IL-1*β*-R: GTTGCTCCATATCCTGTCCCT; Cxcl1-F: AGCCACACTCAAGAATGGTCG, Cxcl1-R: TTACTTGGGGACACCTTTTAG; TNF-α-F: GACGTGGAACTGGCAGAAGAG, TNF-α-R: TTGGTGGTTTGTGAGTGTGAG; Glut1-F: GCCTGACCTTCGGATATGAGC, Glut1-R: TGCCATAGCAGTCAATGAGGA; Hk2-F: TGATCGCCTGCTTATTCACGG, Hk2-R: AACCGCCTAGAAATCTCCAGA; Gpi1-F: TCAAGCTGCGCGAACTTTTTG, Gpi1-R: GGTTCTTGGAGTAGTCCACCAG; Aldoa-F: CGTGTGAATCCCTGCATTGG, Aloda-R: CAGCCCCTGGGTAGTTGTC; Tpi1-F: CCAGGAAGTTCTTCGTTGGGG, Tpi1-R: CAAAGTCGATGTAAGCGGTGG; Gapdh-F: AGGTCGGTGTGAACGGATTTG, Gapdh-R: TGTAGACCATGTAGTTGAGGTCA; Ldha-F: TGTCTCCAGCAAAGACTACTGT, Ldha-R: GACTGTACTTGACAATGTTGGGA; Pdk1-F: GGACTTCGGGTCAGTGAATGC, Pdk1-R: TCCTGAGAAGATTGTCGGGGA. Primers used to detect mouse pancreas purinergic signaling molecules were adopted according to previous reports (14).

### Ethics Statement

All experimental protocols were approved by the Council on Animal Care at Jiangsu University on the Protection and the Welfare of Animals and followed the National Institutes of Health of China guidelines for the care and use of experimental animals.

### Statistical Analysis

Data are presented as mean ± standard deviation (SD). Reproducibility was ensured by performing more than three independent experiments. All statistics were carried out using GraphPad Prism 7.0. Student’s t test was used to compare the differences between two groups. One-way analysis of variance (ANOVA) and post Tukey’s test were used for the three or more groups comparisons. A value of *P <*0.05 was considered to be statistically significant.

## Results

### Expression Profiles of Purinergic Signaling Molecules in Human and Mouse Pancreas Tissue

**Figure 1:**
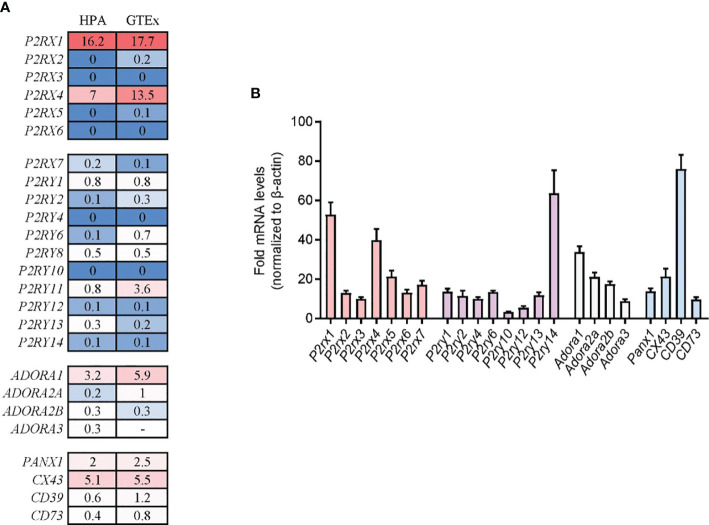
Expression profiles of purinergic signaling molecules in human and mouse pancreas tissue. **(A)** Purinergic signaling molecule expression in human pancreas tissues was analyzed in Human Protein Atlas (HPA) and Genotype-Tissue Expression (GTEx) human tissue RNA sequencing (RNA-seq) databases. Values were presented as protein-coding transcripts per million (pTPM). **(B)** Purinergic signaling molecules expression in mouse pancreas tissues was determined by RT-qPCR and normalized to *β*-actin (n = 4 per group).

The systemic distribution of purinergic signaling molecules in pancreas tissues has not been identified. To investigate the expression profiles of purinergic signaling molecules in pancreas tissues, two nucleotide release channels (PANX1 and CX43), two ectonucleotidases (CD73 and CD39), seven P2X purinergic receptors (P2RX1–7), eight P2Y purinergic receptors (P2RY1/2/4/6/11/12/13/14), and four P1 purinergic receptors (ADORA1/A2A/A2B/A3R) were screened in public Human Protein Atlas (HPA) and Genotype-Tissue Expression (GTEx) human tissue RNA sequencing (RNA-seq) databases. Both databases contain large sample sizes of normal pancreas tissues. We found that the expression profiles of purinergic signaling molecules were quite similar in the two databases. Results showed that most nucleotides release channels, ectonucleotidases, and purinergic receptors could be detected in pancreas tissues (**Figure 1A**), indicating that purinergic signaling was functional in pancreas tissues. Among the purinergic molecules, P2RX2, P2RX3, P2RX5, P2RX6, P2RY4, and P2RY10 were barely detected in one or both databases. In comparison, P2RX1, P2RX4, and ADORA1 were highly expressed in both databases, with P2RX1 being the highest. Next, purinergic signaling molecules were analyzed in mouse pancreas tissues. Generally, the expression profiles of purinergic signaling in mouse pancreas were similar to that of human pancreas. P2RX1, P2RX4, and ADORA1 were also expressed at high levels in mouse pancreas (**Figure 1B**). Therefore, the conversed expression of purinergic signaling molecules in human and mouse pancreas indicates that purinergic signaling system may play crucial roles in regulating pancreas functions.

### Inhibition of Purinergic Signaling Relieves Acute Pancreatitis

**Figure 2:**
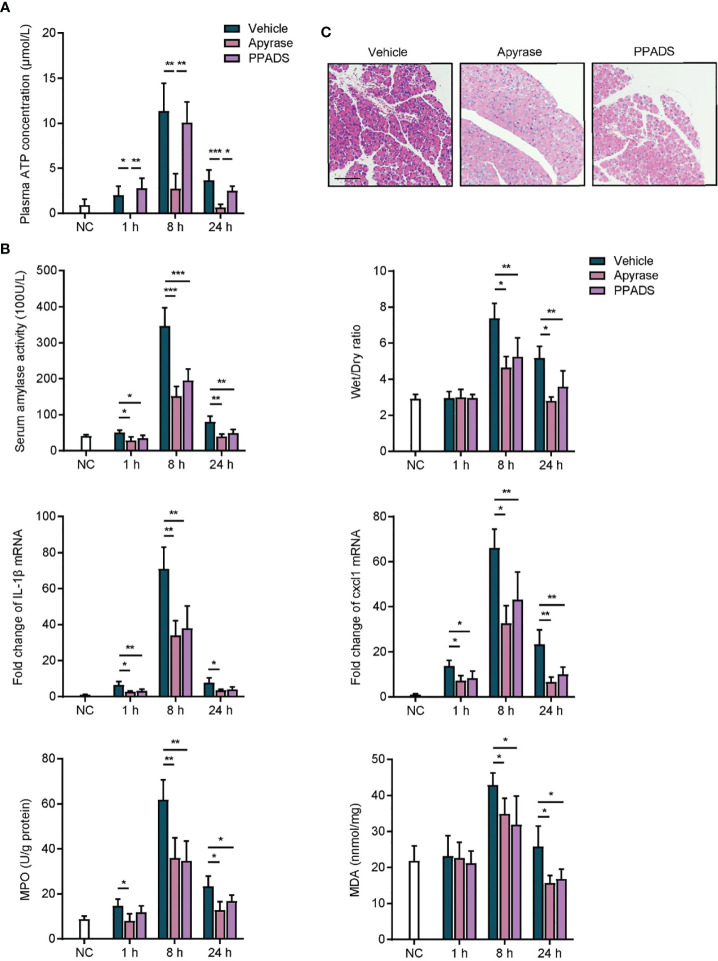
Inhibition of purinergic signaling relieves acute pancreatitis. **(A)** caerulein (50 μg/kg) was intraperitoneally injected seven times hourly to induce mice acute pancreatitis. Apyrase (50 U/kg), PPADS (5 mg/kg) or vehicle was intraperitoneally injected immediately after the first injection of caerulein. Saline-treated mice were set as negative control (NC). Plasma was obtained at 1, 8, and 24 h after the first injection of caerulein, and ATP concentration was detected (n = 5 per group). **(B)** At 1, 8, and 24 h after caerulein exposure, serum amylase activity, pancreas wet/dry ratio, IL1-*β* mRNA expression, Cxcl1 mRNA expression, myeloperoxidase (MPO) activity, and malondialdehyde (MDA) content were analyzed to evaluate inflammatory status (n = 5 per group). 2^−^ *^δδ^* ^Ct^ value was used for comparisons of the fold change of mRNA expression to NC mice. **(C)** Pancreas tissues from each group were harvested at 8 h, stained with H&E and observed under a microscope (100×). Representative images were shown (n = 5 per group). Scale bar is 50 μm. Statistics were calculated using one-way ANOVA with Tukey post-tests. *P< 0.05, **P < 0.01, ***P < 0.001.

AP is an inflammatory disorder of the pancreas, and the etiological factors involved in the initiation and aggravation of AP are still poorly understood (4). Given that purinergic signaling plays crucial roles in inflammatory conditions (15), we sought to determine whether purinergic signaling was involved in the inflammatory responses of AP. Caerulein was intraperitoneally injected by seven hourly doses to induce mice AP. An extracellular ATP hydrolyzing enzyme, apyrase, and a non-specific purinergic receptor antagonist, PPADS, were used to terminate purinergic signaling. Vehicle was used as control. We observed that ATP concentration was significantly increased in the plasma of caerulein-induced AP mice after AP induction (**Figure 2A**), indicating that purinergic signaling was agitated. Owing to the hydrolytic activity, apyrase but not PPADS suppressed the increase of ATP concentration in caerulein-AP mice. Next, inflammatory status in AP mice was evaluated. Serum amylase activity was significantly reduced in mice treated with apyrase and PPADS as compared to vehicle, indicating that pancreas injury was relieved when purinergic signaling was inhibited (**Figure 2B**). Caerulein-induced AP is characterized by an extensive edema, which can be quantified by wet/dry ratio of pancreas tissues. Results showed that wet/dry ratio was significantly increased in vehicle treated mice (**Figure 2B**). Both apyrase and PPADS inhibited the increase of wet/dry ratio, which suggested that pancreas edema was suppressed. Expressions of inflammatory cytokines IL-1*β* and Cxcl1, leukocyte infiltration marker myeloperoxidase (MPO) and lipid peroxidation marker malondialdehyde (MDA) were also reduced by apyrase and PPADS (**Figure 2B**). In addition, hematoxylin eosin (H&E) staining showed that pancreas tissues were significantly injured after caerulein exposure (**Figure 2C**), whereas apyrase and PPADS protected against the pathological damages. Therefore, these results suggest that inhibiting purinergic signaling is sufficient to relieve inflammatory responses in caerulein-induced mice AP.

### Targeting Purinergic Receptor P2RX1 Alleviated Inflammatory Responses in Acute Pancreatitis

**Figure 3:**
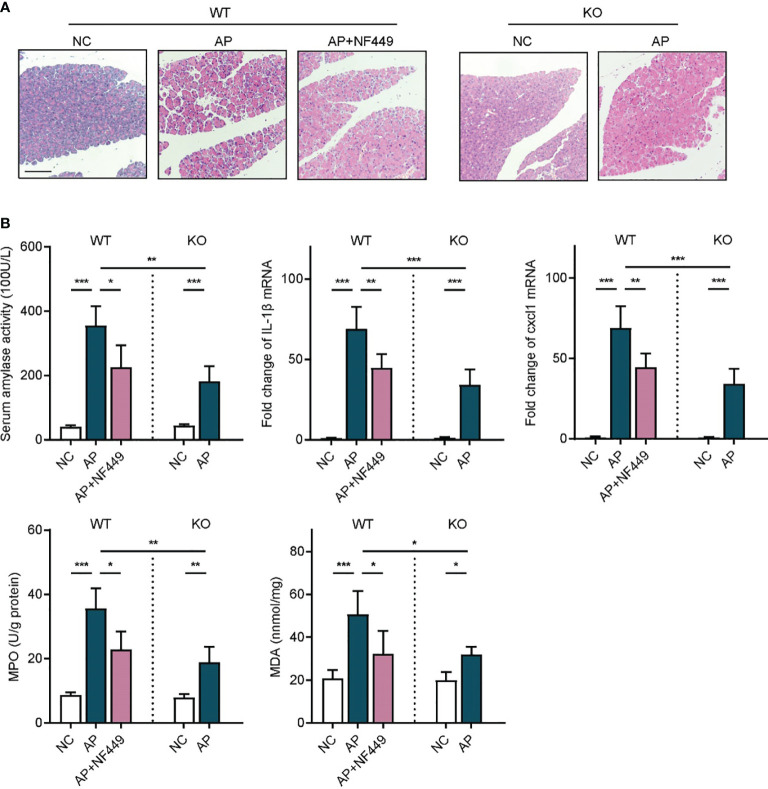
Targeting purinergic receptor P2RX1 alleviated inflammatory responses in acute pancreatitis. **(A)** Caerulein (50 μg/kg) was intraperitoneally injected seven times hourly to induce acute pancreatitis in WT and P2RX1-KO mice. NF449 or vehicle was intraperitoneally injected immediately after the first injection of caerulein. Saline-treated mice were set as negative control (NC). Pancreas tissues from each group were harvested at 8 h, stained with H&E, and observed under a microscope (100×). Representative images were shown. Scale bar is 50 μm. **(B)** At 8 h after caerulein exposure, serum amylase activity, pancreas IL1-*β* mRNA expression, Cxcl1 mRNA expression, myeloperoxidase (MPO) activity, and malondialdehyde (MDA) were analyzed to evaluate inflammatory status (n = 5 per group). 2^−^ *^δδ^* ^Ct^ value was used for comparisons of the fold change of mRNA expression to NC mice. Statistics were calculated using one-way ANOVA with Tukey post-tests. *P < 0.05, **P < 0.01, ***P < 0.001.

Given that apyrase and PPADS are both non-specific inhibitors of purinergic signaling, we next sought to explore the specific purinergic molecule that might be involved in the onset of inflammatory responses in AP. We noticed that P2RX1 was among the most highly expressed purinergic signaling molecules in both human and mouse pancreas tissues (**Figure 1**). However, whether purinergic receptor P2RX1 can mediate the course of AP is unknown. Therefore, a specific antagonist of P2RX1, NF449, was administrated in caerulein-induced mice AP. Notably, we observed that pathological conditions, serum amylase activity, Cxcl1, IL-1*β*, MPO, and MDA were substantially alleviated when mice were treated with NF449 (**Figures 3A, B**). Next, P2RX1 knockout (KO) mice were generated to further evaluate the anti-inflammatory effects of P2RX1. No significant differences of random blood glucose (WT 5.42 ± 0.34 *vs* P2RX1-KO 5.36 ± 0.49 mmol/L, *P* > 0.05) and serum amylase (WT 42.64 ± 3.47 *vs* P2RX1-KO 43.81 ± 6.5 100 U/L, *P* > 0.05) were observed between P2RX1-KO and WT mice. These results indicate that P2RX1 has little regulatory role in pancreas endocrine and exocrine functions. Interestingly, when we compared inflammatory indicators between WT and P2RX1-KO AP mice, results showed that in line with P2RX1 antagonist, P2RX1 genetic ablation markedly relieved inflammatory status in caerulein-induced mice AP (**Figures 3A, B**). Therefore, results from specific antagonist and genetic ablation indicate that purinergic receptor P2RX1 may be a potential therapeutic target in AP.

### Neutrophil-Expressed P2RX1 Contributes to the Progression of Acute Pancreatitis

**Figure 4:**
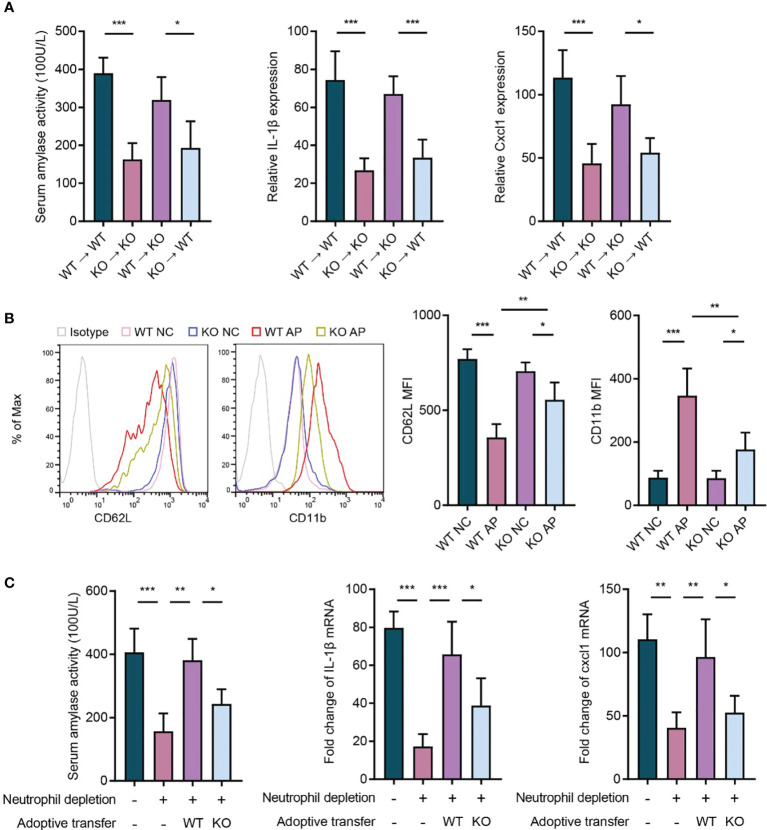
Neutrophil-expressed P2RX1 contributes to the progression of acute pancreatitis. **(A)** Caerulein (50 μg/kg) was intraperitoneally injected seven times hourly to induce acute pancreatitis in bone marrow chimeras (WT → WT, KO → KO, WT → KO, and KO → WT). At 8 h after caerulein exposure, serum amylase activity, pancreas IL1-*β*, and pancreas Cxcl1 expression were analyzed to evaluate inflammatory status (n = 4 per group). **(B)** Caerulein (50 μg/kg) was intraperitoneally injected seven times hourly to induce acute pancreatitis in WT and P2RX1-KO mice. Saline-treated mice were set as negative control (NC). At 8 h after caerulein exposure, flow cytometry was performed to detected CD62L and CD11b expression in blood neutrophils (n = 5 each group). **(C)** Neutrophils in WT mice were depleted by intraperitoneal injection of anti-Ly6G antibody (1A8) 24 h before the first injection of caerulein. Bone marrow neutrophils isolated from of WT or P2RX1-KO mice were intravenously injected 1 h before the first administration of caerulein. At 8 h after caerulein exposure, serum amylase activity, pancreas IL1-*β*, and pancreas Cxcl1 mRNA expressions were analyzed to evaluate inflammatory status (n = 4 per group). 2^−^ *^δδ^* ^Ct^ value was used for comparisons of the fold change of mRNA expression to NC mice. Statistics were calculated using one-way ANOVA with Tukey post-tests **(A, C)**, or Student’s t test **(B)**. *P< 0.05, **P < 0.01, ***P < 0.001.

To further elucidate whether the hematopoietic cell- or non-hematopoietic cell-expressed P2RX1 contributed to the progression of AP, bone marrow chimeras were generated using WT mice and P2RX1-KO mice (WT → WT, KO → KO, WT → KO, and KO → WT). We noticed that AP severity was significantly suppressed in the chimeric mice reconstituted with P2RX1-KO bone marrow cells than those reconstituted with WT bone marrow cells (**Figure 4A**). The results suggested that hematopoietic cell-expressed P2RX1 might promote the inflammatory responses in AP. Among the various types of bone marrow-derived inflammatory cells, neutrophils are considered to play central roles in the pathogenesis of AP. In parallel to previous reports (16), we also found that neutrophils were extensively activated in WT AP mice, as indicated by shedding of CD62L and high expression of CD11b (**Figure 4B**). However, neutrophil activation was suppressed in the neutrophils of P2RX1-KO mice. It suggested that P2RX1 might be required for neutrophil activation in AP. To further confirm the role of neutrophil-derived P2RX1 in AP, WT or P2RX1-KO neutrophils were adoptively transferred to neutrophil-depleted mice, and caerulein was used to induce AP. Results showed that neutrophil depletion could accomplish AP severity (**Figure 4C**). In addition, suppressed inflammatory responses were observed in mice adoptively transferred with P2RX1-KO neutrophils as compared to WT neutrophils (**Figure 4C**). These experiments indicate that neutrophil-expressed P2RX1 is required for the inflammation in AP.

### P2RX1 Is Required for Facilitating Neutrophils’ Activation and Glycolytic Metabolism in Acute Pancreatitis

**Figure 5:**
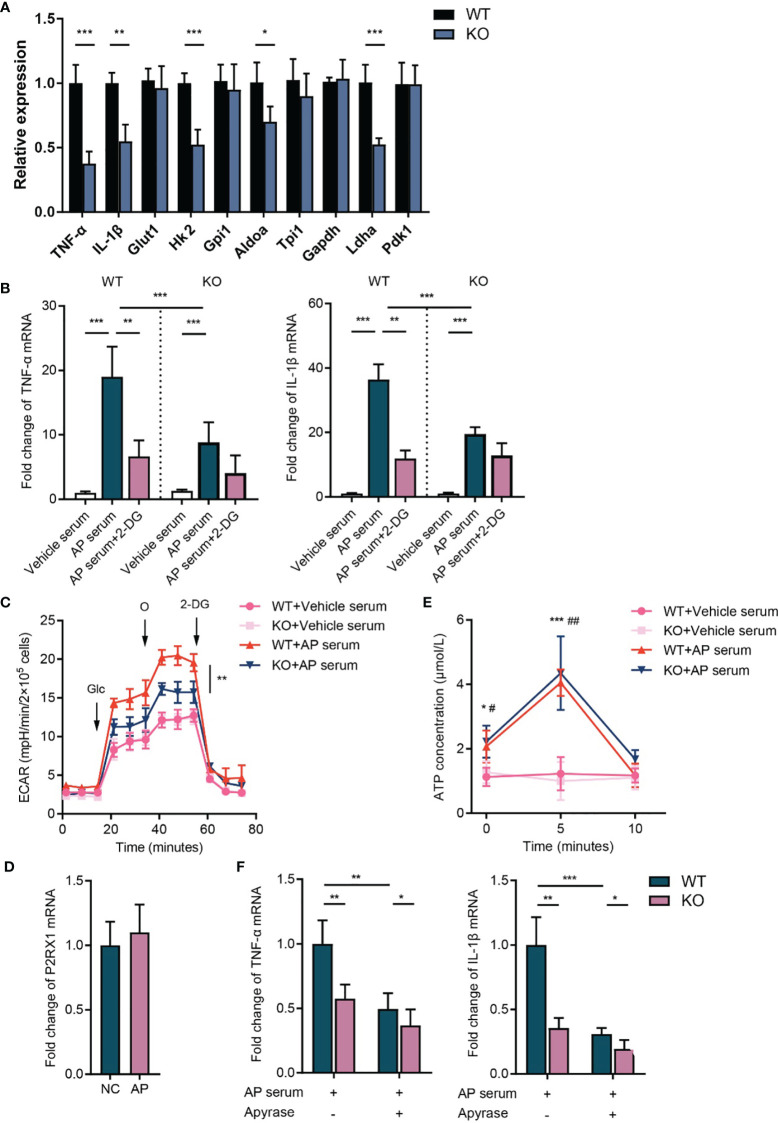
P2RX1 is required for facilitating neutrophil’ activation and glycolytic metabolism in acute pancreatitis. **(A)** Neutrophils were isolated from pancreas of WT or P2RX1-KO mice at 8 h after the first administration of caerulein. Expressions of inflammatory cytokines and glycolytic metabolism genes were detected by RT-qPCR. 2^−^ *^δδ^* ^Ct^ value was used for comparisons the fold change of mRNA expression to WT neutrophils. **(B)** Neutrophils were isolated from bone marrow of unstimulated WT or P2RX1-KO mice. Serum was used to stimulated neutrophils in the presence or absence of 2-DG for 6 h. Inflammatory cytokines were measured by RT-qPCR. 2^−^ *^δδ^* ^Ct^ value was used for comparisons the fold change of mRNA expression to vehicle serum-treated WT neutrophils. **(C)** Neutrophils were isolated from bone marrow of WT or P2RX1-KO mice. After stimulation with AP mice serum for 6 h, extracellular acid ratio (ECAR) was measured. **(D)** Pancreas P2RX1 expression in NC mice or AP mice were determined by RT-qPCR. 2^−^ *^δδ^* ^Ct^ value was used for comparisons of the fold change of mRNA expression to NC pancreas. **(E)** Neutrophils were isolated from bone marrow of WT or P2RX1-KO mice and stimulated with AP serum. Extracellular ATP was detected in indicated time points. **(F)** Neutrophils were isolated from bone marrow of WT or P2RX1-KO mice and stimulated with AP serum with or without apyrase. Inflammatory cytokines were measured by RT-qPCR. 2^−^ *^δδ^* ^Ct^ value was used for comparisons of the fold change of mRNA expression. Statistics were calculated using Student’s t test **(A, E, F)**, or one-way ANOVA with Tukey post-tests **(B, C)**. *P < 0.05, **P < 0.01, ***p < 0.001.

Recently, accumulating evidence reveals that cellular metabolic reprogramming has direct roles in mediating immune cell function, and enhanced glycolysis supports the activation of inflammatory cells (17). However, whether the metabolic shift contributes to P2RX1-induced neutrophil activating in AP remains unknown. To address this issue, neutrophils were isolated from inflammatory pancreas tissues, and expressions of inflammation- and metabolism-associated genes were detected. Results showed that expression of IL-1β and TNF-α was significantly increased in WT neutrophils comparing to P2RX1-KO neutrophils (**Figure 5A**). Interestingly, a similar expression pattern was observed in glycolysis-associated genes (**Figure 5A**), indicating that WT neutrophils had enhanced inflammatory status and glycolytic metabolism. Next, naïve neutrophils were isolated from unstimulated WT and P2RX1-KO mice and then activated by AP mice-derived serum. We observed that basal inflammatory cytokines were similar between WT and P2RX1-KO neutrophils (**Figure 5B**). However, a more intense increase of IL-1*β* and TNF-α was obtained in WT neutrophils than in P2RX1-KO neutrophils after stimulation with AP serum. Next, extracellular acid ratio (ECAR) was detected to evaluate glycolytic metabolism (**Figure 5C**). We found that ECAR was similar in unstimulated WT and P2RX1-KO neutrophils. AP serum stimulation significantly increased ECAR in both WT and P2RX1-KO neutrophils, whereas a higher ECAR was observed in WT neutrophils. It suggests that although P2RX1 is essential for supporting neutrophil glycolysis, other P2RX1-independent pathways still exist. To further investigate whether enhanced glycolysis in WT neutrophils accounted for neutrophil activation, 2-Deoxy-D-Glucose (2-DG), an inhibitor of hexokinase II was used to block glycolytic pathway. Results showed that 2-DG markedly blocked IL-1*β* and TNF-α expression, and the IL-1*β* and TNF-α level in WT neutrophils were reduced to a similar level to that of P2RX1-KO neutrophils (**Figure 5B**). These results indicate that P2RX1-involved glycolytic metabolism may contribute to neutrophil activation in AP.

Next, we sought to determine the mechanism of neutrophil P2RX1 activation in AP. We noticed that P2RX1 expression was not altered in the pancreas of AP mice (**Figure 5D**). Considering that P2RX1 is a fast-responding ATP-gated ion channel, we speculate that the time-consuming transcriptional regulation may not be involved in the activation of neutrophil P2RX1 in acute inflammation. Previously, we found that Gram-negative bacteria-derived lipopolysaccharide-induced autocrine ATP activated neutrophils within 10 min (18). To explore whether the autocrine release of ATP facilitates neutrophil activation in AP, we detected ATP release in neutrophils treated with AP serum. We observed that ATP concentration was significantly increased in the supernatant of WT neutrophils at 5 min after AP serum activation (**Figure 5E**). Genetic knockout of P2RX1 had no effect on neutrophil ATP release. Then, autocrine-released ATP, as well as AP serum-derived ATP were hydrolyzed by apyrase, and we observed that TNF-α and IL-1*β* expression was significantly reduced in WT neutrophils and which was less apparent in P2RX1-KO neutrophils (**Figure 5F**). These results suggest that neutrophil P2RX1 can be activated by the rapidly autocrine-released ATP or the peripheral context-derived ATP in AP.

## Discussion

AP is a common gastrointestinal disorder with a poor clinical outcome (19). The mechanisms of overactivated inflammatory responses in AP are still undefined, and the treatment of AP is limited to supportive therapy (20, 21). ATP is an indispensable energy currency inside mammalian cells. Under steady conditions, extracellular ATP concentration is barely detected owing to the presence of ectoapyrases and ectoadenosine triphosphatases (18). However, under stress conditions, intracellular ATP is readily released into extracellular compartments and the concentration may rise from ∼10 nM to 5–8 mM (22). By activating P2 purinergic receptors, extracellular ATP is involved in the pathogenesis of various inflammatory diseases such as transplantation rejection and autoimmune disease (23). Nevertheless, little is known about the role of purinergic signaling in AP.

By investigating the expression profiles of purinergic signaling-associated molecules in human and mouse pancreas tissues, we found that P2RX1 was among the most highly expressed genes in both species, suggesting that P2RX1 might play important roles in regulating pancreas function. However, the specific effect of P2RX1 is unclear. Therefore, we generated P2RX1-KO mice and studied whether P2RX1 could mediate inflammatory responses in AP. Results showed that inflammatory factors and pancreas injuries were markedly suppressed in P2RX1-KO mice. Interestingly, usage of the specific P2RX1 antagonist achieved a similar effect as genetic ablation of P2RX1, suggesting the therapeutic potential of targeting P2RX1 in AP.

Existing reports indicate that several P2 purinergic receptors, including P2X1R, P2X7R, P2Y2R, and P2Y14R, are expressed in neutrophils, and recent studies have highlighted the essential role of purinergic signaling in neutrophil activation (24). Activated by extracellular ATP, purinergic receptor(s)-involved signaling is initiated and which further regulates neutrophil functions, including transmigration (25), chemotaxis (13), neutrophil extracellular traps (26), oxidative burst (27), and degranulation (28). Neutrophils are considered as the first responders in the acute phase of AP, and substantial activation of neutrophils is a hallmark of disease aggravation (29). Given that ATP concentration is highly upregulated in AP, we speculate that P2RX1-involved neutrophil activation may contribute to inflammation of AP. To investigate the specific cellular resource of P2RX1, we performed bone marrow chimeras and adoptive transfer studies. Results uncovered that neutrophil-expressed P2RX1 is essential for inflammatory responses in pancreas tissues. Our further *in vivo* and *in vitro* experiments showed that P2RX1 was essential for neutrophil activation in AP.

Studies in recent decades have confirmed that immune cell functions are finely regulated by cellular metabolism, and glycolysis mainly characterizes and supports immunostimulatory phenotypes (30). In the present study, we found that P2RX1-KO neutrophils expressed less glycolytic genes and had lower glycolytic metabolism as compared to WT neutrophils. In parallel, inflammatory cytokines were also reduced in AP serum-activated P2RX1-KO neutrophils. Blocking glycolytic metabolism by 2-DG, inflammatory cytokines in WT neutrophils were markedly abolished to a similar level as P2RX1-KO neutrophils. It was noticed that glycolysis and inflammatory cytokine expression were both slightly upregulated in activated P2RX1-KO neutrophils as compared to naïve neutrophils, indicating that other pathways might also participate in neutrophil activation. Moreover, inflammatory cytokines and damage-associated molecular patterns that are liberated in caerulein-induced sterile inflammation are able to promote neutrophils mediated release of ATP (31), which may further amplify the occupying of P2RX1 signaling and set off a feed-forward loop of neutrophil activation.

In conclusion, our study shows that neutrophil-expressed P2RX1 contributes to the inflammatory responses of AP by inducing a glycolytic metabolism-involved mechanism. Purinergic signaling molecules are abundantly expressed in pancreas tissues, with P2RY2, P2RY4, and P2RY6 shown to regulate *β* cell secretion, and P2RY2, P2RY4, and P2RY11 are found to regulate pancreatic ducts’ secretion (32). Whether these P2 receptors can influence the progression of AP is still unknown, which merits additional investigations. In addition, it is worth noting that caerulein-induced murine AP is an *in vivo* model of mild AP, and whether our findings relate to severe AP or clinical AP needs further verification.

## Data Availability Statement

The original contributions presented in the study are included in the article/supplementary material. Further inquiries can be directed to the corresponding authors.

## Ethics Statement

The animal study was reviewed and approved by Council on Animal Care at Jiangsu University on the Protection and the Welfare of Animals.

## Author Contributions

XW, DZ, WQ, YL and XY performed experiments. DL and XZ analyzed the data, and designed figures. XW, DZ, and CD conceived the idea and wrote the manuscript. All authors contributed to the article and approved the submitted version.

## Funding

This study was supported by the National Natural Science Foundation of China (ID: 81701945 to XW); China Postdoctoral Science Foundation (ID 2018M640403 to XW).

## Conflict of Interest

The authors declare that the research was conducted in the absence of any commercial or financial relationships that could be construed as a potential conflict of interest.
